# Photocatalytic Degradation of Tetracycline by a Novel (CMC)/MIL-101(Fe)/β-CDP Composite Hydrogel

**DOI:** 10.3389/fchem.2020.593730

**Published:** 2021-01-12

**Authors:** Hui Zhang, Liang Zhou, Jing Li, Sijia Rong, Jianping Jiang, Shengquan Liu

**Affiliations:** School of Forestry and Landscape Architecture, Anhui Agricultural University, Hefei, China

**Keywords:** photo-catalytic, MIL-101(Fe), β-CDP, tetracycline, hydrogel

## Abstract

Herein, we report a novel carboxymethyl cellulose (CMC)/MIL-101 (Fe)/poly(β-cyclodextrin) (β-CDP) hydrogel with high photocatalytic activity. β-CDP can significantly enhance the photoactivity of MIL-101(Fe) in the hydrogel prepared by a simple solvothermal method. The structure and property of this composite hydrogel were characterized by Fourier transform infrared spectroscopy, X-ray diffraction, scanning electron microscopy, and transmission electron microscopy. Tetracycline was selected as a model pharmaceutical antibiotic to evaluate the photocatalytic activity of the composite hydrogel under visible light irradiation and darkness, respectively. This composite hydrogel shows excellent activity for degrading pharmaceutical antibiotics under visible light irradiation. The increased photocatalytic activity can be attributed to β-CDP, which acts as a promoter and affords an efficient separation of photogenerated electron-hole pairs of MIL-101(Fe). Moreover, the composite hydrogel is shown to have good water retainability. The hydrogel is inexpensive and shows high photocatalytic activity. Hence, it can be used as an efficient photocatalytic material.

## Introduction

Antibiotics are widely used to cure livestock of various diseases (Choi et al., 2007). Tetracycline shows a high antimicrobial activity and hence is the second most commonly used antibiotic worldwide (Wang et al., 2011). However, because of its extensive use, it has become a common environmental pollutant and is found in soil, waste systems, and even drinking water (Gartiser et al., 2007). Therefore, various methods have been developed to treat tetracycline, such as chemical oxidation, membrane separation, and photocatalysis (Jiang et al., 2020).

Semiconductor photocatalysts are used to treat contaminated water and air (Ranjbari et al., 2020). At present, some of the most commonly used semiconductor photocatalysts are based on transition-metal oxides, such as TiO_2_, MnO_2_, and ZnO (Sun and Lin, 2009). However, a major problem with these photocatalysts is that they have a large band gap and, hence, can be used only with ultraviolet irradiation. Therefore, it is important to design and develop photocatalysts that can be used under visible light (Xuan and Xiao, 2012; Zhang et al., 2014).

Metal organic frameworks (MOFs) have attracted a significant interest of the research community over the last two decades (Furukawa et al., 2010). MOFs are used in a wide variety of applications, such as sensing, gas separation, and photocatalysis, because of their excellent properties, such as high porosity and high surface area. Moreover, in contrast to the traditional semiconductor photocatalysts, the absorption bands of MOFs can be adjusted to the visible light because they contain a large amount of organic linkers (Araya et al., 2017; Yao et al., 2018; Wang et al., 2019; Chen et al., 2020; Li et al., 2020b). In addition, a composite of adsorption-enrichment and *in-situ* photocatalytic degradation of MOFs may greatly improve the pollutant removal performance (Wang et al., 2020).

Hydrogels have attracted more and more interests in water pollution treatment because of their large specific surface area, strong adsorption, and a large amount of exposed active sites. Cellulose-based hydrogels have become potential research directions because of their biocompatibility and biodegradability. However, a major drawback in the preparation and application of these hydrogels is the weak capacity to load hydrophobic substances (Chen et al., 2017).

β-Cyclodextrin (β-CD), one of the most widely used host molecules, exhibits good affinity for various organic compounds. Because of its hydrophobic cavity, β-CD can be encapsulated to form inclusion complexes with a wide range of hydrophobic molecules for improving its poor hydrosolubility because of its hydrophilic surface and hydrophobic cavity (Tian and Liu, 2020). It has recently been reported that poly(β-cyclodextrin) (β-CDP) with high specific surface areas can be obtained by reacting β-CD with epichlorohydrin (EPI) in an alkaline medium. The resulting insoluble polymers can be reused for the separation of organic pollutants and dyes (Uzqueda et al., 2011; Zhou et al., 2019).

Herein, we report a novel carboxymethyl cellulose (CMC)/MIL-101(Fe)/β-CDP composite hydrogel with potential application in the photocatalytic oxidation of tetracycline under ambient conditions. The structure and properties of the obtained hydrogel composite were characterized by Fourier transform infrared spectroscopy (FTIR), X-ray diffraction (XRD), scanning electron microscopy (SEM), and transmission electron microscopy (TEM).

## Materials and Methods

### Materials

2-Aminoterephthalic acid (NH_2_-BDC) was purchased from Sigma-Aldrich Co. LLC (Shanghai, China). Ferric trichloride hexahydrate and dimethyl sulfoxide were purchased from Sinopharm Chemical Reagent Co., Ltd. (Shanghai, China). Carboxymethyl cellulose sodium (CMC), tetracycline, EPI (AR), phenolphthalein (AR), and β-CD (1,135 g mol^−1^, purity >98%) were obtained from Aladdin Industrial Co., Ltd. (Shanghai, China). All other chemicals were of analytical grade, obtained from commercial suppliers, and used without further purification unless otherwise noted.

### Synthesis of MIL-101(Fe)

MIL-101(Fe) was prepared by reacting NH_2_-BDC (0.45 g) with FeCl_3_·6H_2_O (1.35 g) in the presence of dimethyl sulfoxide (30 ml) at 110°C for 24 h. The orange solid was recovered by filtration, washed with hot water and ethanol, and finally dried overnight at 150°C under vacuum.

### Synthesis of β-CDP

First, 10 g of β-CD was dissolved in 15 ml of 15 wt.% NaOH at room temperature. After magnetically stirring the solution for 24 h, 2 ml of toluene was added and stirred for 120 min at 35°C. Then, 6.89 ml of EPI was added with vigorous stirring for 200 min. The mixture was dissolved with water and neutralized with 6 mol L^−1^ of hydrochloric acid. The resulting solution was dialyzed for 7 days (MWCO 8–12 kDa; Biosharp, USA) and recovered by freeze drying to obtain a white product.

### CMC/MIL-101(Fe)/β-CDP Hydrogel Preparation

An aqueous sodium hydroxide solution of CMC/MIL-101(Fe)/β-CDP was prepared using 1.5 g of CMC, 0.001 g of MIL-101(Fe), and 1.0 g of β-CDP. Then, 10 ml of this solution was stirred for 30 min. Next, 1 ml of EPI was added to the CMC/MIL-101(Fe)/β-CDP solution, reacted for 60 min at room temperature, and then kept at 50°C for 5 h to obtain the composite hydrogel. And, a CMC/MIL-101(Fe)/β-CD with 1.0 g of β-CD was prepared by the same method as a comparative experiment.

### Characterization

The FTIR spectra of the CMC/MIL-101(Fe)/β-CDP hydrogel were collected with a Bruker Tensor II Fourier transform infrared spectrometer. The test specimens were prepared by the KBr-disc method. The phase structure and purity of the samples were examined by XRD (MAP18XAHF) using a diffractometer with Cu Kα radiation (λ = 1.54 Å) at a scanning rate of 2°/min. The morphology and microstructure were characterized by a SEM instrument (Hitachi S-4800, Japan). The fractured surfaces of the samples were sputtered with gold before observing and photographing them. The UV–vis absorbance of the samples was measured using a UV-1800 spectrophotometer (Shimadzu, Japan).

### Swelling Experiments

The swelling capacity of two CMC/MIL-101(Fe)-based hydrogels of β-CD and β-CDP with time was investigated. The experiments were performed using a definite quantity (about 0.1 g) of the materials, which were transferred to previously weighed tea bags and then placed in a beaker containing 100 ml of distilled water to attain equilibrium at room temperature. The samples were collected from the distilled water at regular intervals (30 min). Before recording the weights of the hydrogels, their surfaces were wiped with a filter paper to remove distilled water. And, the swelling capacity of two CMC-based hydrogels of β-CD and β-CDP without MIL-101(Fe) was investigated by the same method as a comparative experiment.

### Photocatalytic Tests

The photocatalytic activity of the samples was evaluated based on the selective oxidation of tetracycline under visible light irradiation obtained using a 500 W xenon lamp. In a typical process, 10 mg of each CMC/MIL-101(Fe)-based hydrogel of β-CD and β-CDP was dispersed in 100 ml of an aqueous solution of 50 mg/L of tetracycline. The resulting mixture was stirred for 30 min before performing the test. After several hours of irradiation, the mixture was centrifuged at 12,000 rpm to completely remove the photocatalyst. The reactive oxygen species (ROS) generated from the samples under irradiation was determined by decomposing 1,3-diphenylisobenzofuran (DPBF) at 410 nm and by measuring the decomposition using a UV-1800 spectrophotometer. And, photocatalytic test of 0.001 g of MIL-101(Fe) was prepared by the same method as a comparative experiment. In order to ascertain the reusability of the composite hydrogels, degradation experiments were investigated. In each recycling run, hydrogel of CMC/MIL-101(Fe)/β-CDP was recycled after alternately washing three times with water and ethanol and drying in a vacuum oven at room temperature for 24 h.

## Results and Discussion

**Figure 1:**
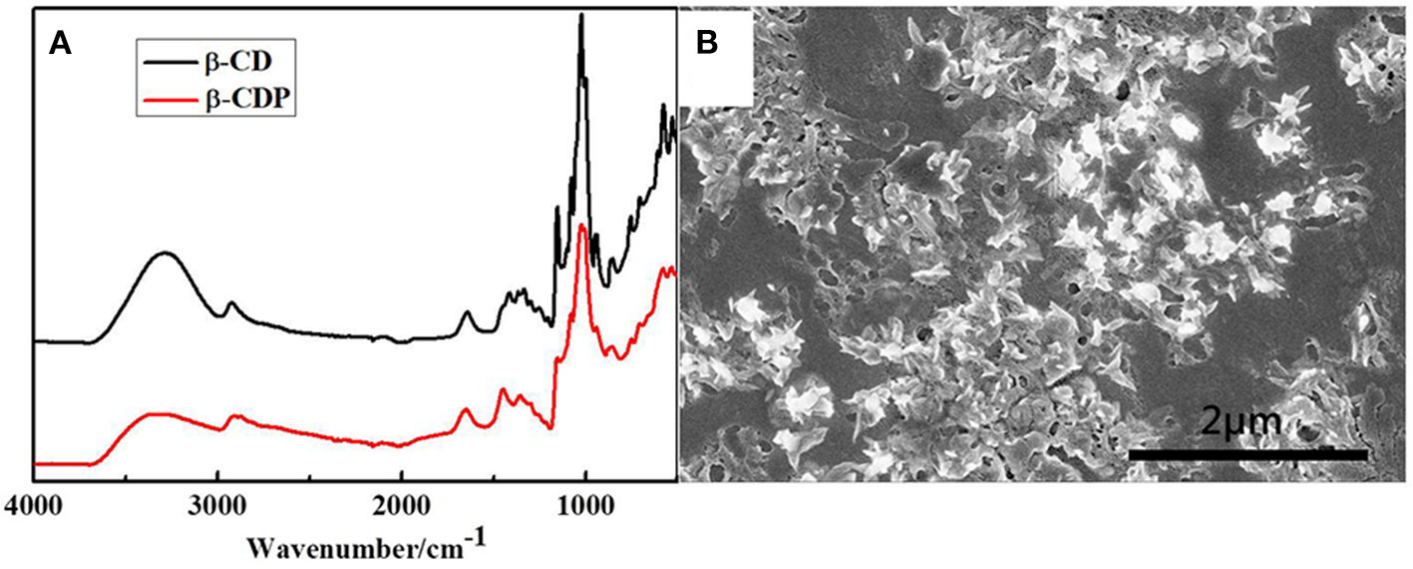
FTIR spectra of β-CD and β-CDP **(A)** and SEM image of β-CDP **(B)**.

The FTIR spectra of β-CD and β-CDP are shown in Figure 1. The band at 2,922 cm^−1^ corresponds to the –CH_2_ stretching vibration of the samples. The O–H bending vibration of β-CDP at 3,446 cm^−1^ decreased, and its C–O–C stretching vibrations at 1,033 cm^−1^ increased in comparison with that of β-CD. This phenomenon occurs because of the breakage and rearrangement of hydrogen bonds during the polymerization of β-CD (Sun et al., 2017). After the polymerization, β-CDP forms a fairly large sheet of ~20 nm (Figure 1B), which indicates that β-CD molecules are cross-linked with EPI.

**Figure 2:**
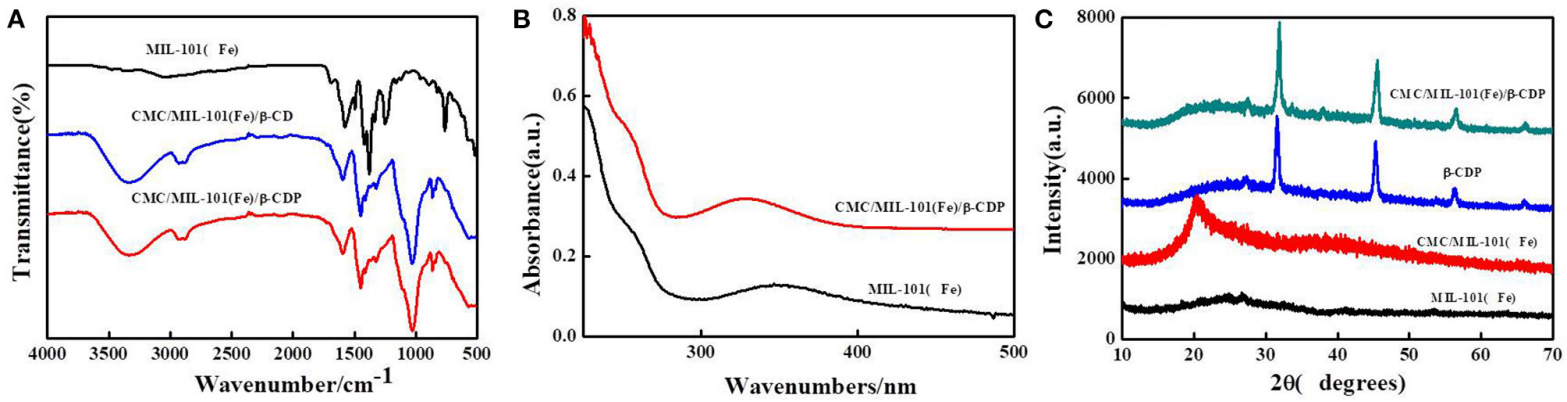
FTIR spectra of MIL-101(Fe), CMC/MIL-101(Fe)/β-CD, and CMC/MIL-101(Fe)/β-CDP **(A)**, UV–vis spectra of MIL-101(Fe) and CMC/MIL-101(Fe)/β-CDP **(B)**, and XRD images of MIL-101(Fe), CMC/MIL-101(Fe), β-CDP, and CMC/MIL-101(Fe)/β-CDP **(C)**.

To analyze the molecular structure of the composite hydrogel, the FTIR spectra of MIL-101(Fe) and β-CD and β-CDP hydrogels are shown in Figure 2. For the MIL-101(Fe) sample, the sharp peaks at 1,603 and 1,393 cm^−1^ are assigned to the asymmetric and symmetric stretching vibrations of O–C–O in carboxyl groups, respectively. The band at 756 cm^−1^, attributed to the C–H bending vibration, is characteristic of benzene rings. The band at 542 cm^−1^ is ascribed to the stretching vibration of the Fe–O band in the MIL-101(Fe) framework (Deng et al., 2018). In the composite hydrogels of β-CD and β-CDP, the absorption peaks at 1,602, 1,393, 756, and 542 cm^−1^ are attributed to MIL-101(Fe). The UV–vis absorption spectra of MIL-101(Fe) show photo-adsorption at wavelengths of 225 and 348 nm. The UV–vis adsorption spectra of (CMC)/MIL-101(Fe)/β-CDP composite hydrogel show a slight blue shift from 348 to 330 nm. The crystallographic structure of MIL-101(Fe) and that of the as-prepared CMC/MIL-101(Fe)/β-CDP composite hydrogel were determined by powder XRD. The collected diffraction pattern of MIL-101(Fe) at 2θ = 24.65° matched well with the reported pattern (Duan et al., 2018), indicating that MIL-101(Fe) had been successfully prepared. And the characteristic peaks of CMC/MIL-101(Fe) and β-CDP were also shown in Figure 2C. The collected diffraction pattern of CMC at 2θ = 21°, β-CDP, and composite hydrogel showed similar sharper diffraction peaks. The XRD results also prove successful fabrication of the CMC/MIL-101(Fe)/β-CDP composite hydrogel without impurities.

**Figure 3:**
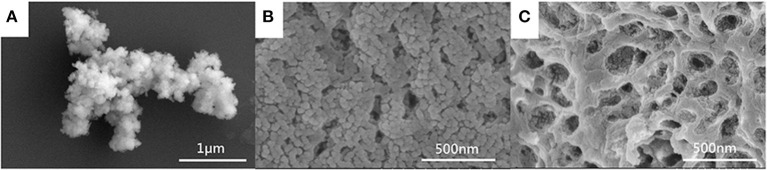
SEM images of MIL-101(Fe) **(A)** and CMC/MIL-101(Fe) composite hydrogels with β-CD **(B)** and β-CDP **(C)**.

The morphology of the MIL-101(Fe) and CMC/MIL-101(Fe) composite hydrogels with β-CD and β-CDP was observed using SEM. The as-prepared MIL-101(Fe) displays a spherical structure of size ranging from 200 to 300 nm (Figure 3A). Interpenetrating porous and network structures of both hydrogels are clearly visible in Figures 3B,C respectively. It can be observed that the network structure comprising β-CDP was denser, whereas the hydrogel made of β-CD was looser. The pore diameters of both the β-CD and β-CDP composite hydrogels ranged from 100 to 200 nm. Therefore, materials with smaller pores can be used as guest materials in these hydrogels to obtain a larger specific surface area, which facilitates the uptake of a large amount of solvent.

**Figure 4:**
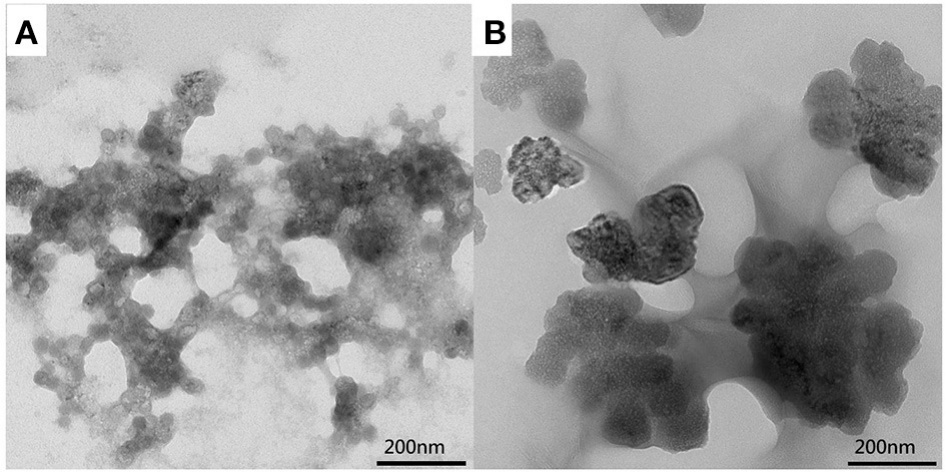
TEM images of the CMC/MIL-101(Fe) composite hydrogel with β-CD **(A)** and β-CDP **(B)**.

The hydrogels were further examined by TEM. The TEM image of the CMC/MIL-101(Fe)/β-CD composite hydrogel is shown in Figure 4A. MIL-101(Fe) is well-dispersed in the hydrogel without agglomeration, which demonstrates that MIL-101(Fe), CMC, and β-CD were combined very well in the composite. The CMC/MIL-101(Fe)/β-CDP composite hydrogel (Figure 4B) is dense and has high strength, probably due to a higher degree of cross-linking. However, some hydroxyl groups on the β-CDP surface react with the EPI, thereby reducing the moisture content of the hydrogel.

**Figure 5:**
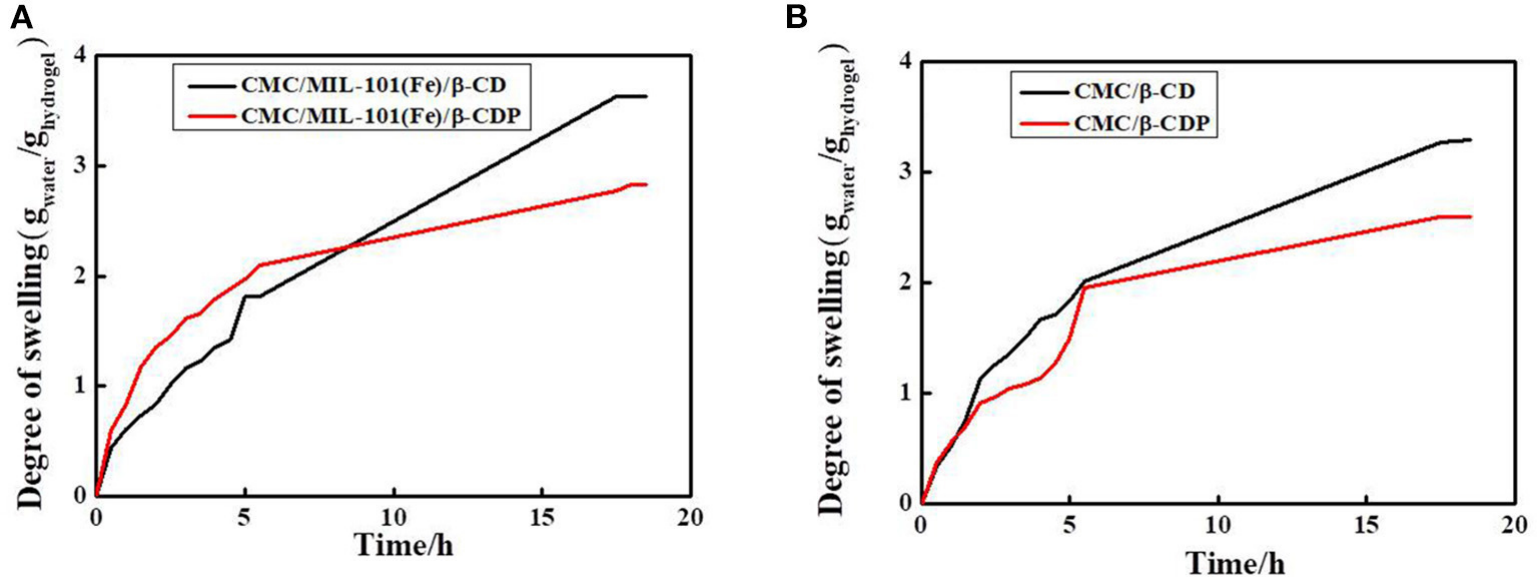
Swelling behavior of the hydrogels. **(A)** CMC/MIL-101(Fe)/β-CD and CMC/MIL-101(Fe)/β-CDP, **(B)** CMC/β-CD, and CMC/β-CDP.

The equilibrium swelling degree is an important parameter to evaluate the property of a hydrogel (Hu et al., 2016). Figure 5 shows the water-uptake curves of CMC/MIL-101(Fe) with β-CD and β-CDP. Both the hydrogels swell rapidly in the beginning, and a swelling balance is obtained after ~20 h. When β-CD was used in the hydrogel, the optimal swelling percentage is obtained, with a swelling percentage of 363%. This result can be ascribed to the relatively open net-structure and a hydrophilic exterior of the CMC/MIL-101(Fe)/β-CD composite hydrogel. However, the hydrogel with β-CDP shows a slight decrease in its water-uptake capacity (283%), probably because the β-CDP surface has fewer hydroxyl groups, as discussed before. The swelling percentage of CMC/β-CD was 330%, and the swelling percentage of CMC/β-CDP was 260%; it indicated that MIL-101(Fe) has little effect in the swelling degree of this composite hydrogel.

**Figure 6:**
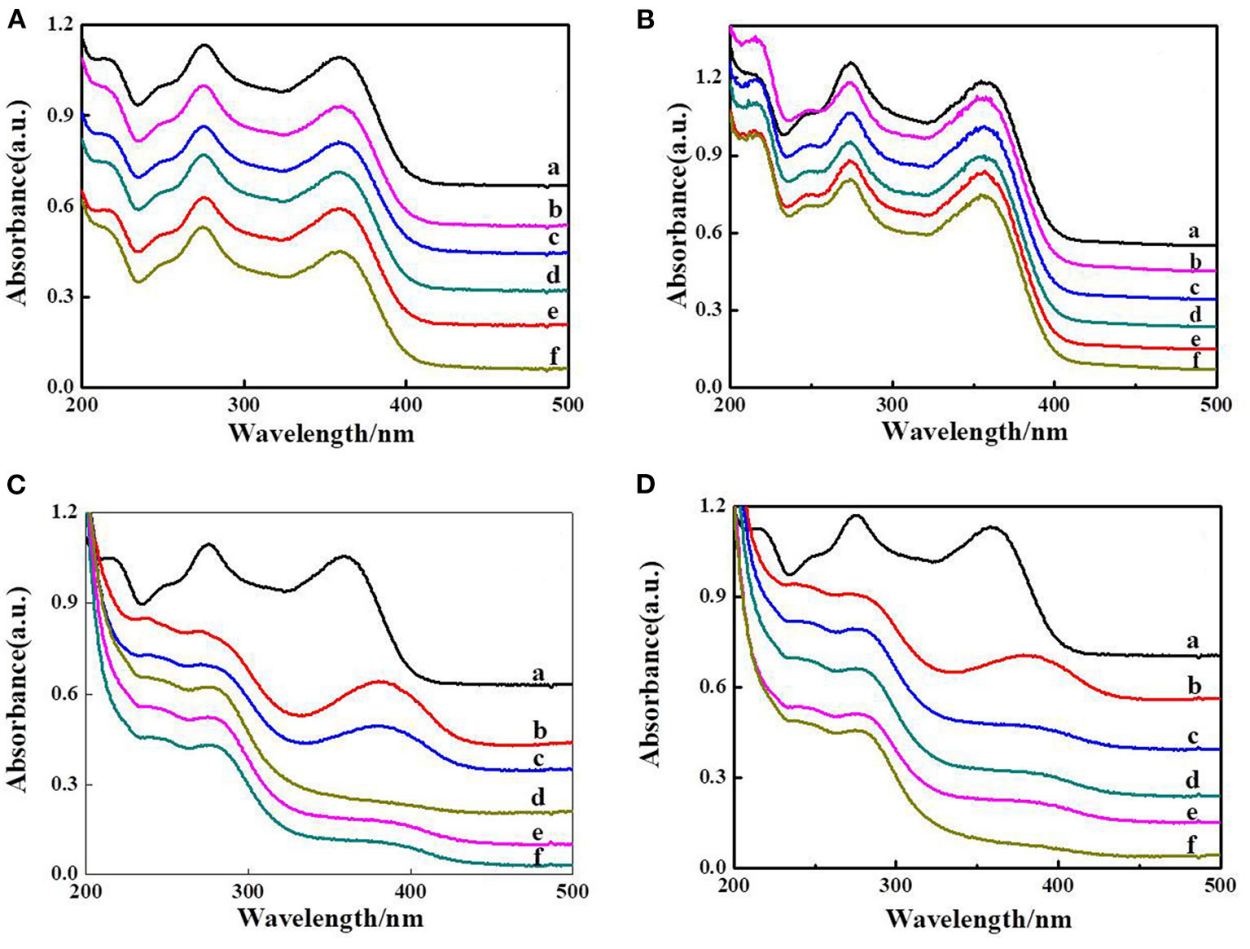
Decomposition of tetracycline, blank group only with irradiation **(A)**, MIL-101(Fe) **(B)**, CMC/MIL-101(Fe) composite hydrogel with β-CD **(C)**, and β-CDP **(D)**; a–f means that the times of irradiation were 0.5, 1, 1.5, 2, and 2.5 h.

The photocatalytic activity of the as-prepared composite hydrogels was studied using tetracycline as the target contaminant under visible light irradiation. Control experiments were also carried out. The results showed that tetracycline is resistant to degradation under visible light irradiation only (Figure 6A). We can also observe that a little tetracycline was degraded with MIL-101(Fe) (Figure 6B); it may be only a small amount of MIL-101(Fe) that had poor dispersion in the tetracycline solution [0.001 g of MIL-101(Fe) added in 500 ml of tetracycline solution]. The overall activities of different hydrogels are also compared using the same amount of the samples (10 mg). About 40% tetracycline canbe degraded by composite hydrogel of CMC/MIL-101(Fe)/β-CD after in 30 min under visible light (Figure 6C). Approximately 85% of tetracycline was degraded by the CMC/MIL-101(Fe)/β-CDP composite hydrogel in 30 min (Figure 6D). This excellent photocatalytic performance of the CMC/MIL-101(Fe)/β-CDP hydrogel is owing to the hydrophobic inner cavity of β-CDP and the acceleration of the charge transfer rate from MIL-101(Fe) to the electron acceptors.

**Figure 7:**
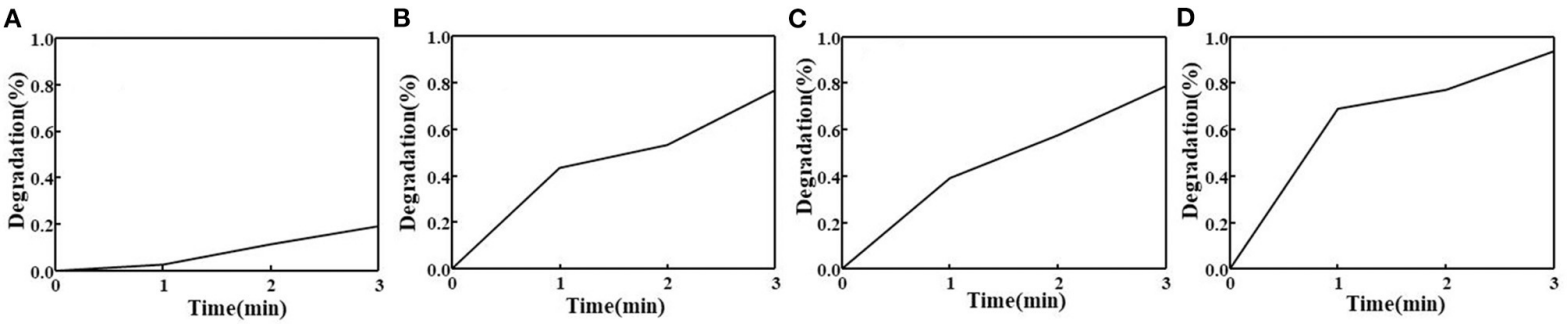
Photodecomposition of DPBF by ROS upon the irradiation of pure DPBF **(A)**, pure MIL-101(Fe) **(B)**, and composite hydrogel with β-CD **(C)** and β-CDP **(D)**.

To evaluate the capability of MIL-101(Fe) in generating ROS in the composite hydrogel under visible light irradiation, DPBF was used as a detector. ROS determined by the decomposition of DPBF correlated well with the decay of absorption at 410 nm (Zhang et al., 2017). Only 19% of DPBF decomposed under visible light within 3 min (Figure 7A). Figure 7B shows the ROS output of freshly prepared MIL-101(Fe) as a function of visible light irradiation time. Approximately 77% of DPBF decomposed within 3 min, reflecting a very high ROS yield. The ROS output of the composite hydrogel with β-CD showed a slight decrease compared with that observed for the hydrogel with β-CDP; the former showed 79% degradation of DPBF (Figure 7C), and the latter showed 94% degradation (Figure 7D). Such a high degradation was observed probably because the hydrogel network with β-CDP shows an appreciable encapsulation capacity for MIL-101(Fe).

**Figure 8:**
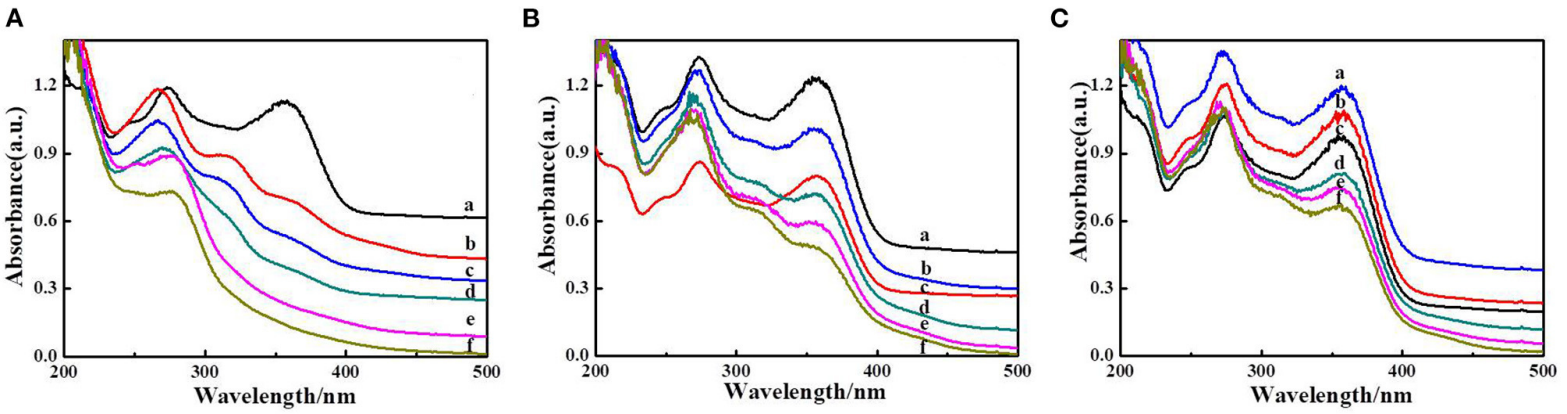
Photodecomposition of tetracycline by CMC/MIL-101(Fe)/β-CDP composite hydrogel after **(A)** 1, **(B)** 2, and **(C)** 3 days.

In order to ascertain the reusability of the hydrogel CMC/MIL-101(Fe)/β-CDP, degradation experiments were investigated. In each recycling run (Figure 8), the hydrogel with better catalytic performance for the photocatalytic degradation of tetracycline during 2.5 h indicated that the CMC/MIL-101(Fe)/β-CDP could be reused during the degradation reaction. The photocatalytic activity of the hydrogel decreases slightly after three successive runs can be attributed to the adsorption capacity of hydrogel is getting to saturation state along with photocatalytic process, which blocked up the 3D porous structure of hydrogel and limited photonic efficiency.

**Figure 9:**
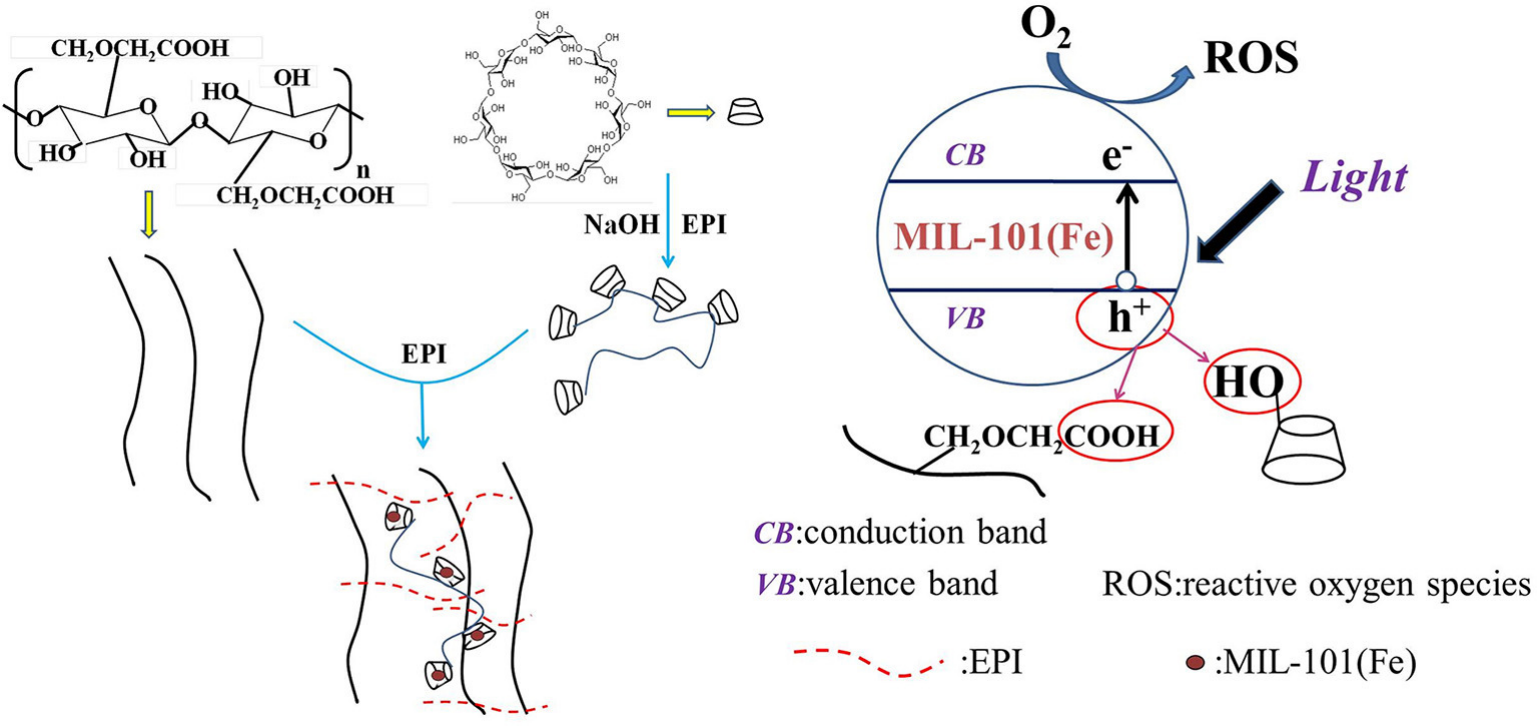
Proposed photocatalytic mechanism in CMC/MIL-101(Fe)/β-CDP composite hydrogel.

The high photocatalytic performance of composite hydrogel could be attributed to the high surface area, absorption capability, and separation efficiency of photoinduced charge carriers (He et al., 2020; Li et al., 2020a). The possible photocatalytic mechanism in composite hydrogels was depicted in Figure 9. MIL-101(Fe) was located in the cavities of β-CDP. As long as visible light irradiation started, the MIL-101(Fe) could be activated to yield electrons in the conduction band and holes in the valence band. The hydroxyl and carboxymethyl of β-CDP and CMC could trap the photogenerated holes resulting in the lower e^−^/h^+^ recombination.

## Conclusions

A novel CMC/MIL-101(Fe)/β-CDP composite hydrogel with a high photocatalytic activity was developed. The as-prepared MIL-101(Fe) displays good photocatalytic ability under visible light irradiation. The β-CDP supporting hydrogel matrix remarkably improves the photostability and photocatalytic efficiency of MIL-101(Fe) for the selective oxidation of tetracycline. In addition, the CMC/MIL-101(Fe)/β-CDP composite hydrogel has a simple preparation process, low cost, and high photocatalytic effect. Hence, the CMC/MIL-101(Fe)/β-CDP hydrogel composite system is a highly promising catalyst for use in the field of environment engineering.

## Data Availability Statement

The original contributions presented in the study are included in the article/supplementary materials, further inquiries can be directed to the corresponding author/s.

## Author Contributions

The experiment design and manuscript preparation were done by HZ with LZ. JL investigated the morphology and crystal structure of the composite hydrogel. SR and JJ investigated the photocatalytic performance of composite hydrogel. SL supervised the work and together with HZ wrote the publication. All authors contributed to the article and approved the submitted version.

## Conflict of Interest

The authors declare that the research was conducted in the absence of any commercial or financial relationships that could be construed as a potential conflict of interest.
